# Slow-moving soil organisms on a water highway: aquatic dispersal and survival potential of Oribatida and Collembola in running water

**DOI:** 10.1186/s40462-019-0165-5

**Published:** 2019-07-02

**Authors:** Meike M. Schuppenhauer, Ricarda Lehmitz, Willi E. R. Xylander

**Affiliations:** 1 Senckenberg Museum of Natural History Görlitz, Am Museum 1, 02826 Görlitz, Germany; 2 Faculty of Biology, Technische Universität Dresden, 01062 Dresden, Germany; 3 International Institute Zittau - TU Dresden, Markt 23, 02763 Zittau, Germany

**Keywords:** Passive transport, Hydrochory, Submersion tolerance, Colonisation, Long-distance dispersal, Survival rate

## Abstract

**Background:**

Oribatida and Collembola are an important part of the soil food web and increase soil fertility by contributing to the recycling of nutrients out of dead organic matter. Active locomotion enables only limited dispersal in these tiny, wingless arthropods, while passive dispersal plays an important role for long-distance dispersal. Previous investigations have focused on passive transport by wind, other animals, or sea currents, whereas studies on transport via running water are missing. However, previous observation of the long survival of submerged terrestrial microarthropods makes passive dispersal with running water very likely.

**Methods:**

By combining field and lab experiments, we studied the potential for passive dispersal of oribatid mites with running water. We investigated terrestrial Oribatida and Collembola: (1) along a stream taking soil and moss samples, (2) in a stream using sticky covers and aquarium nets, and (3) studied their ability to colonise new soil after aquatic transport with the help of floating islands. Furthermore, we investigated the survival of submerged Oribatida species and their floating capabilities in lab experiments to predict dispersal distances with running water. We tested for differences between species using Kruskal-Wallis test for equal medians and Mann-Whitney pairwise-comparison and χ^2^-test for the influence of body size on aquatic dispersal.

**Results:**

Soil and moss samples revealed a pool of 52 oribatid mite species at the stream bank. Within the stream, we caught 180 individuals from 36 oribatid mite species. Only 14 of those species were also found in the soil and moss samples, whereas the remaining 22 were of unknown origin. Based on material caught on sticky covers, an average of 63.9 (± 54.6) oribatid mite individuals fell on one m^2^ stream water per week. Four species of Collembola (27 individuals) and 21 species of oribatid mites (47 individuals) were collected with aquarium nets. Eight microarthropod species (Oribatida + Collembola) successfully colonised new soil in the floating islands after aquatic dispersal. Lab experiments showed that Oribatida can float for at least 14 hours at the surface of running water and may survive for more than 365 days when submerged. The floating abilities and survival rates were largely species-specific.

**Conclusion:**

This is the first study to demonstrate successful passive dispersal with running water for two groups of terrestrial soil microarthropods, including subsequent colonisation of new soil. We show that submersion survival, as well as floating abilities, and therefore dispersal capability, are not only high in oribatid mites, but also species-specific. Running waters obviously serve as long-distance dispersal highways for many of these less mobile soil-living animals.

**Electronic supplementary material:**

The online version of this article (10.1186/s40462-019-0165-5) contains supplementary material, which is available to authorized users.

## Background

Dispersal is specified as any movement of an organism or propagules, potentially influencing spatial gene flow [1]. While active dispersal of an organism can be directed, passive dispersal results in an undirected way of distribution and requires forces external to the individual organism [2]. Although dispersal can be risky [3], it may also increase an individual’s fitness [4]. Understanding species ranges and predicting the future distribution of invasive species requires detailed knowledge about dispersal mechanisms [5, 6]. For studies on dispersal in larger animals, such as birds and mammals, GPS-tracking or mark-and-recapture can be applied. However, studying the dispersal capacity of small arthropods living hidden in the soil is much more difficult, but not less important [7].

Soil is an extremely complex system with a huge species diversity [8–10], characterized by a strongly structured, three-dimensional space composed of a litter layer and the soil pores [11]. The soil ecosystem is characterized by a high spatial heterogeneity as a result of biotic interactions within pore spaces and variation in soil architecture at the microscale and by variation in chemical and physical properties in the range of centimetres to meters [10]. The complexity, the tremendous biodiversity and opacity of soil, are some reasons why our ecological understanding of belowground organisms is still limited, although they play a key role in maintaining functioning ecosystems and aboveground communities [12–14].

The two most widespread, abundant, and ecologically important groups of soil microarthropods are oribatid mites (Oribatida) and springtails (Collembola). They can be found in various types of terrestrial habitats and are most abundant in the first centimetres of humus rich soil, with up to 300,000 individuals of oribatid mites per m^2^ in temperate forests [15, 16]. Also, Collembola are most abundant in soil and can be found down to 150 cm below the surface [17]. Some oribatid mite and Collembola species are arboricoles, colonising lichens and moss covers or the bark of trees [18–20]. Only a few oribatid species spend all stages of their life cycle submerged in freshwater [21], while aquatic Collembola species live on the surface of the water (pneustonic), but not truly subaquatic [19]. In many parts of the world, Oribatida and Collembola have never been studied and a large number of species have yet to be discovered. For oribatid mites, about 11,000 species have been described worldwide [22] and around 560 of them occur in Germany [20]. Nevertheless, it is estimated that five to ten times more species of Oribatida are still unknown [21]. In Collembola, there are around 6500 species known to science, and 420 of them occur in Germany [17, 23]. Oribatid mites are between 0.2 and 2.0 mm in size [24], while Collembola range from 0.1 to 10 mm [17, 23]. Their life-span is commonly one to two years in temperate regions and may span five or more years under cold conditions [17, 25–28].

Oribatida and Collembola are a relevant part of the soil food web and influence soil fertility [29]. By consumption of different food sources, spanning between three and four trophic levels [30–33], they support decomposition, fungal dispersal, and nutrient cycling [30, 34]. In newly exposed and developing environments, such as glacier forelands, mining pits, and dumps, soil microarthropods belong to the first colonizers that modify the primary habitat conditions and promote succession [35–41]. Furthermore, many microarthropod species have huge geographical ranges, with some having worldwide distributions [19, 24, 42]. Therefore, efficient dispersal pathways must exist, but they are still poorly understood. Active dispersal can only play a minor role for long-distance migration because most soil microarthropod species cover only short distances through their own locomotive power [43, 44]. Particularly, oribatid mites are highly limited in their dispersal ability, even in distances of only five centimetres, while springtails show no limitation in the range of 300 cm [45]. However, passive dispersal by larger animals [46, 47] and by wind [39, 48, 49] may allow relatively rapid long-distance transport. Also, transport of terrestrial microarthropods overseas has been suggested [50] and examined once [51], but direct observation is difficult. Concerning running water, published evidence of aquatic dispersal of soil microarthropods is lacking. Instead, studies mainly focus on survival rates on and underwater to predict aquatic dispersal and subsequent colonisation [52, 53]. Nevertheless, passive dispersal of soil organisms in running water is very likely, as oribatid mites and Collembola can survive submerged up to 165 and 15 days, respectively [52, 54]. Carabid beetles survived for one to two weeks on the surface of freshwater and a maximum of twelve days underwater at about 10 °C [55], but underwater survival was reduced to only two to three days at elevated temperatures of 16 to 18 °C. Myriapod species survived for 65 days submerged in 10 °C freshwater [56] or 72 days in freshwater of 15 °C [57]. Further studies have reported physiological, morphological and behavioural adaptations of soil organisms to flood situations, like plastron respiration, enclosure in an air bubble, or a lowered metabolic rate [58–61]. These adaptations would also facilitate the survival of organisms during aquatic transport.

**Fig. 1:**
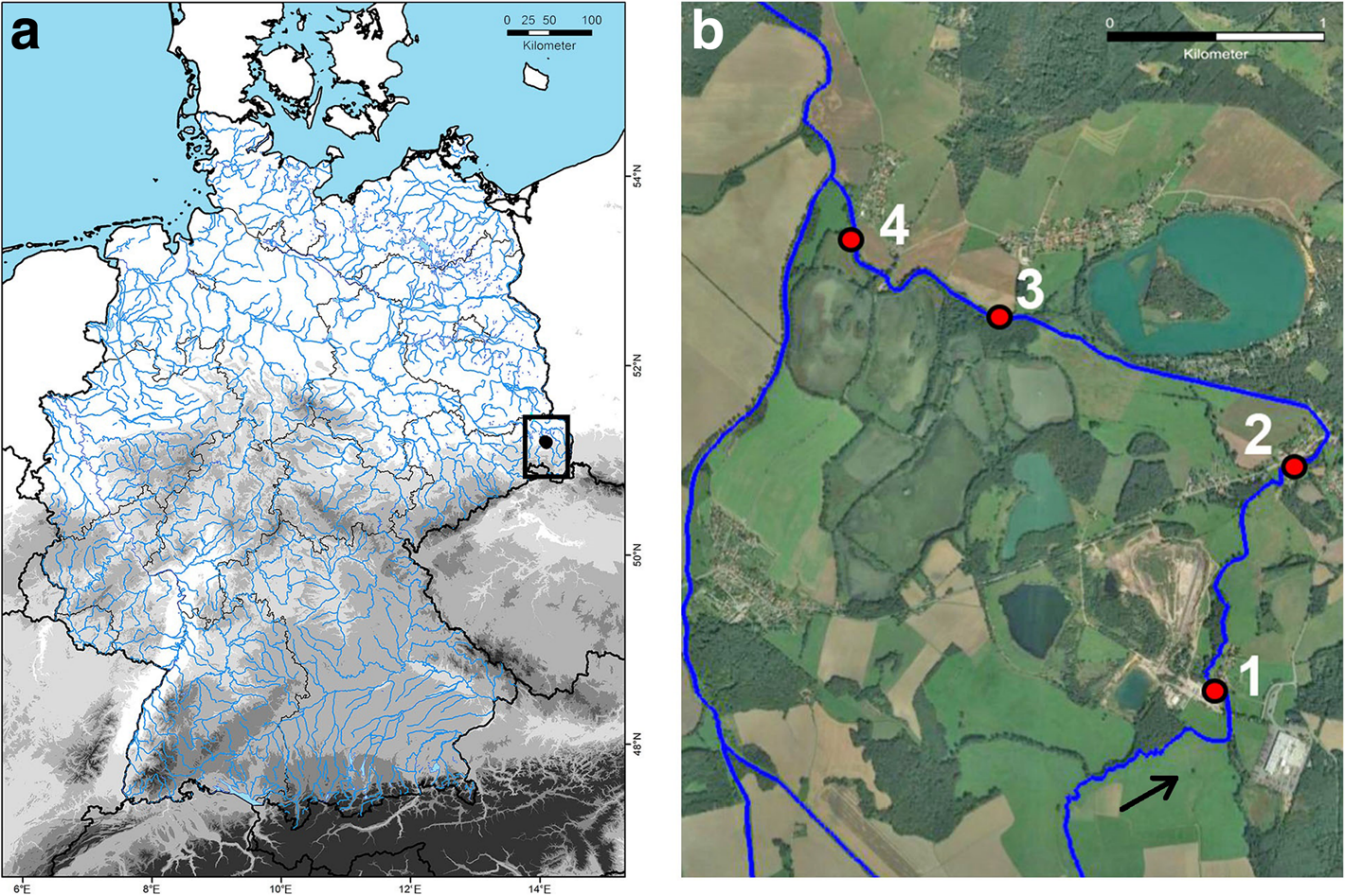
**a** Map of German running waters with position of study site. **b** Sampling sites at the Altes Fließ (arrow indicates flow direction)

Rivers and streams traverse whole countries and may provide transport for less mobile organisms. In Germany, for instance, all running waters, including brooks and rivers, comprise a total length of about 400,000 km [62] (Fig. 1). Microarthropods settle in high abundances on floodplains and river banks and may accidentally be swept away. Between 3000 and 37,000 ind./m^2^ of oribatid mites can be found in habitats temporarily flooded by a river [63]. In soil with about 30% water content next to a river, 17,000 ind./m^2^ of Oribatida were found [64]. For Collembola, between 4072 ind./m^2^ in wet floodplain grasslands and 12,674 ind./m^2^ in fresh floodplain grasslands were observed [65]. Flooding may reduce the abundance of Collembola by about 4.6 times compared to a control site [66], but densities can increase again after flooding from 6-11,000 ind./m^2^ to 10-19,000 ind./m^2^ within six weeks [67].

Considering the survival rates of arthropods, as well as their abundances on freshwater banks and floodplains, we can assume that soil microarthropods are regularly passively dispersed along rivers. However, to our knowledge, no published study has demonstrated waterborne dispersal of soil animals by running waters and subsequent colonisation of new soil. Accordingly, information about the percentage of riverbank species that is transported via water is missing. In the present study, we therefore focused on the following questions: (1) Which terrestrial oribatid mite species occur along running waters? (2) Which species of Oribatida and Collembola can be found in running water and in what numbers? (3) Are they able to colonise new soil after aquatic transport? (4) How long can oribatid species float on water and survive underwater, i.e., what distances can they overcome either on the surface or in water?

## Material and methods

### Site description

For the field study, a small stream called “Altes Fließ” located within and adjacent to the biosphere reserve “Oberlausitzer Heide- und Teichlandschaft”, Saxony, Germany, was selected (Fig. 1; maps were compiled with ArcGIS, Map data: 2018© Google LLC Geo-Basis-DE/BKG and Adobe Photoshop CS6 13.0.1.). Four sampling sites were chosen along the stream. The sites were numbered in flow direction; sampling site 1 (furthest upstream; WGS 84: 51°15′15.8″N, 14°35′35.2″E), followed by 2 (51°15′49.7″N, 14°35′55.7″E), 3 (51°16′13.4″N, 14°34′43.7″E) and 4 (51°16′26.7″N, 14°34′5.5″E). Distances between sampling sites were 1.4 km (1 to 2), 1.9 km (2 to 3) and 1.0 km (3 to 4). The stream crosses agricultural lands, forests, private gardens, and a camping area and has a total length of about 5.9 km. It diverges from the “Dubrauker Fließ” and flows into the “Löbauer Wasser”, which discharges into the Spree River. Within the sampling periods in 2016 and 2017, stream width ranged from 1.7 to 4.5 m, and water depth in the middle of the stream ranged from 22 cm to 45 cm (Table 1). The area is characterized by continental climate [68]. The mean annual air temperature is 10.4 °C, and the mean annual amount of precipitation is 570 mm (Königswartha meteorological station, about 18 km northwest of the sampling sites) [69].

**Table 1:** Mean, minimum, and maximum stream parameters at the four sampling sites in the “Altes Fließ”

sampling site	stream width [m]			water depth [cm]			water temp [°C]			pH			velocity [m/sec]		
	mean	min	max	mean	min	max	mean	min	max	mean	min	max	mean	min	max
1	2.57	2.40	2.90	28.3	22.0	37.0	15.3	9.15	20.3	7.3	6.4	8.5	0.06	0.02	0.09
2	2.06	1.98	2.12	37.1	34.5	39.5	15.2	9.0	21.9	7.4	7.0	7.7	/	/	/
3	4.34	4.17	4.50	33.7	24.0	44.0	14.8	9.0	20.7	7.3	6.2	8.2	0.06	0.04	0.08
4	1.88	1.68	2.17	31.7	22.0	45.0	16.4	15.0	17.8	7.4	6.4	8.5	0.17	0.09	0.25

During sampling, temperature and pH were measured two to three times about 5 cm below the water’s surface at every sampling date and site. Mean water temperature ranged from 14.8 to 16.4 °C (Table 1). Mean pH was between 7.3 and 7.4. Velocity was measured ten times at every sampling site directly beneath the water’s surface (FlowTracker2, SonTek – Xylem Inc.) on 14 and 18 September 2016. Velocity had a mean of 0.1 m/sec.

### Field work

To evaluate the oribatid mite species along the stream and to compare them to the species encountered in the stream, we took soil and moss samples on 18 Oct and 9 Nov 2017. Six soil cores, 32.17 cm^2^ each, were taken with a soil corer to a depth of 5 cm at each of the sampling sites 1 – 3, 1.5 m from the water line. At the same sampling sites, one moss sample was taken from each of three trees, at a height of 10 to 160 cm. In total, 18 soil cores and nine moss samples were transported directly to the lab and were slowly heat extracted from 20 °C to 55 °C with a MacFadyen high-gradient apparatus for ten days, starting on the day of sampling. Microarthropods were captured in 70% Ethanol and Tris-EDTA. All individuals of Oribatida were counted and the adults were identified to species level.

In order to detect aquatic dispersal of oribatid mites and Collembola, we used three sampling methods: aquarium nets, floating islands, and sticky covers [70]. Aquarium nets (JBL, 20 cm width, mesh size 500 μm) were used to capture floating individuals on the water’s surface. Per sampling site, five nets were fixed to a rope, which was attached to a tree at each side of the stream. Distance between adjacent nets was five to 20 cm. The lower half of the net opening was placed under water.

**Fig. 2:**
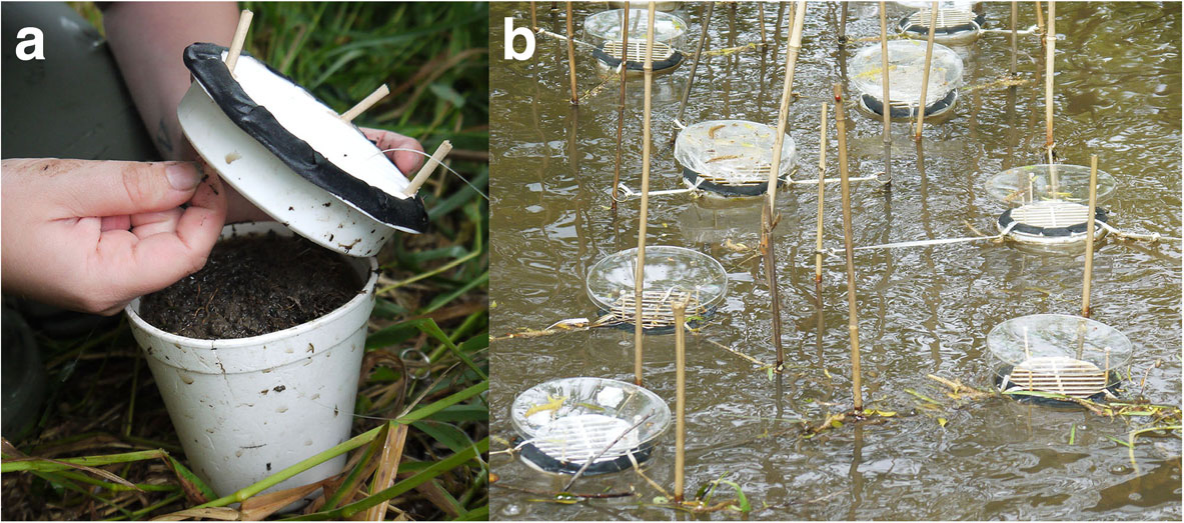
**a** Fixation of wall fan grille to a floating island. **b** set-up of islands at sample site 1

To investigate the survival during aquatic drift and subsequent colonisation potential, we constructed floating islands (9.2 cm height, opening 10.8 cm in diameter; Fig. 2) [70]. These islands were filled with soil, which had been defaunated by heating to 90 °C for 10 min in a microwave and subsequent freezing for seven days at − 20 °C. The islands were closed with a wall fan grille (Fig. 2) and fixed in the stream using three bamboo sticks, rubber bands, and key rings, preventing horizontal drift, but allowing vertical movement of the islands [70]. In order to prevent individuals from landing on the island from the air, each island was covered with a petri dish (diameter: 14 cm) and could therefore only be entered from the stream. Five of these petri dishes per sampling site were covered with insect glue (Aurum Insekten Leim, Neudorff). These sticky covers allowed recording of individuals entering the stream by wind dispersal. Unfortunately, the method was not suitable for the preservation of Collembola, so identification of them was impossible.

Sampling was done over two years: the first sampling period lasted from 10 Aug 2016 to 30 Nov 2016 at three sampling sites (1, 3, 4), second sampling was from 2 May to 11 Oct 2017 (sampling site 1, 2, 3). In 2017, one sampling site from 2016 (number 4) was not accessible anymore; therefore a new sampling site (number 2) was established as a replacement. We set up five aquarium nets and five sticky covers per sampling site, which were exchanged every week for a five week period. Aquarium net catches and sticky covers were examined under a stereomicroscope (Leica M80). In the first sampling year, we installed five floating islands per sampling site. In 2017, we increased the number to 13 per sampling site to compensate for loss of islands over the sampling period. One island per sampling site was taken from the stream after 4, 8, 12, and 16 weeks in 2016. In 2017, we had planned to take out three islands per sampling site every four weeks and therefore installed 39 islands. Unfortunately, due to heavy rain and storms, we lost a large number of islands or the petri dishes covering them in 2017, and only 18 of the 39 islands could be included in the analyses. In detail, we retrieved after 4 weeks: 3 + 3 + 3 islands; after 12 weeks: 0 + 3 + 3 islands, and after 23 weeks: 3 + 0 + 0 islands in 2017 (the three numbers representing the three sampling sites). Soil microarthropods were heat extracted from the islands’ soil as described for the soil samples.

### Lab experiments

To record the floating ability on running water and the survival rate of submerged oribatid mites, we used two experimental designs: (1) floating experiments and (2) submersion experiments. Animals were extracted from additional soil and moss samples taken in the study area next to the stream on 18 Oct and 9 Nov 2017. Specimens were extracted alive with a Berlese-Tullgren apparatus into breeding containers and supplied with a moist plaster of Paris-activated coal mixture. Specimens were kept in two breeding containers at 15 °C in a dark climate chamber and were fed weekly with green algae *Pleurococcus* sp. (SAG Göttingen), dry yeast (Dr. Oetker), and powdered wheatgrass (Flügelschwinger) for a period of two to 16 weeks until the start of the experiments. The temperature of the climate chamber corresponded to the average temperature of the “Altes Fließ”. Only adult mites were used for the submersion and floating experiments.

For the floating experiments, we used an oval-shaped basin of 89 × 51.5 × 9 cm filled with tap water, with a channel width at the surface of 10.3 cm (BIG-Waterplay). The inner surface was painted white using waterproof paint to improve the contrast to the dark mites. A 4.5 V propeller (diameter = 2 cm) was installed at the bottom of the basin to create a mean current velocity of 0.05 m/s in the middle of the channel. Specimens were placed onto the water’s surface and checked hourly for eight to 14 h until they sunk. Floating experiments were performed with six oribatid mite species and four to 12 individuals per species (Fig. 4). Individuals were used only once and were identified to species level after the experiment.

The submersion experiment was adapted to methods described by Coulson et al. 2002 [52] and Pfingstl 2013 [53]. In our case, specimens were kept in 9.4 × 10.7 × 6.8 mm (300 μL) imaging chambers on μ-Slides, with eight chambers per slide, closed with a cover slip (μ-Slide 8 well, ibidi). The slides allowed for a separate observation of single, totally submerged individuals on a small area without any disruption by opening the lid. On the bottom of each chamber, a single layer of wetted milk filter paper (Universal) was placed to enable the specimens to have contact with the substrate. Subsequently, each specimen was separately placed into a chamber, tap water (15 °C) was added, and the lid was closed so no air-bubbles remained under the lid. Submersion experiments were performed with eleven oribatid mite species, between eight and 24 individuals per species (Fig. 5). Species were randomly distributed between the slides to compensate for potential treatment effects. Experiments started stepwise on 28 Nov and 5 Dec 2017 and 11 Jan, 25 Jan, and 09 Mar 2018. The experiment was stopped on 25 Jan 2019, after a maximum period of 365 days underwater. At that point, still living individuals were preserved in 98.5% Ethanol, identified, and included into the statistical analysis.

Slides were observed daily under a stereomicroscope (Leica M80) to record activity of specimens. If an individual was inactive, it was first stimulated with increasing light intensity of the stereomicroscope. If the individual still did not move, the lid was opened and the specimen was touched with a fine brush. If no movement was visible after 30 s, it was taken out, placed onto dry filter paper, and observed for another minute. It was then considered to be dead without any further movement and stored in 70% denatured Ethanol for species identification. In the few cases that an air bubble appeared underneath the lid, the chamber was opened, a few drops of 15 °C tap water were added, and the lid was closed again.

### Species identification and habitat preferences

Prior to identification, specimens were cleared in lactic acid on object slides. Identification to species level was done for all adults using a Leica Microscope (DM2500). For most species, juveniles are not yet described [71], so they were grouped together as ‘Oribatida juveniles’ or ‘Collembola juveniles’. Species identification of Oribatida followed Weigmann 2006 [24], except for the genus *Phthiracarus* Perty, 1841, which was identified using Beck et al. 2014 [72]. Collembola were identified using Fjellberg 1998 [19] and 2007 [42]. Main habitat preferences were taken from Weigmann et al. 2015 [20] for Oribatida and from Fjellberg 1998 & 2007 [19, 42] for Collembola.

### Statistical analysis

To evaluate the influence of the body size class of Oribatida on aquatic dispersal, we used a χ^2^-test. The relationships between body size class and sampled habitats (soil samples vs. moss samples vs. stream (sticky covers + aquarium nets + floating islands)) were analysed. Species were assigned to size classes according to their average body length [24]: < 300 μm, 300–500 μm and > 500 μm. Standardized residuals were examined to evaluate the significant differences between observed and estimated values of two variables (size class and habitat). To test for significant differences between the survival rates of the species kept underwater, we performed a Kruskal-Wallis test for equal medians. For pairwise comparison of species’ survival rates, we used the Mann-Whitney comparison and chose Bonferroni corrected *p*-values. All analyses were performed with PAST 3.23 [73]. Due to the loss of many floating islands and the low number of specimens in the remaining islands, we were not able to do any statistics on that part of the study, but decided to include these results nevertheless as proof of principle (colonisation after aquatic dispersal).

## Results

### Field work

**Fig. 3:**
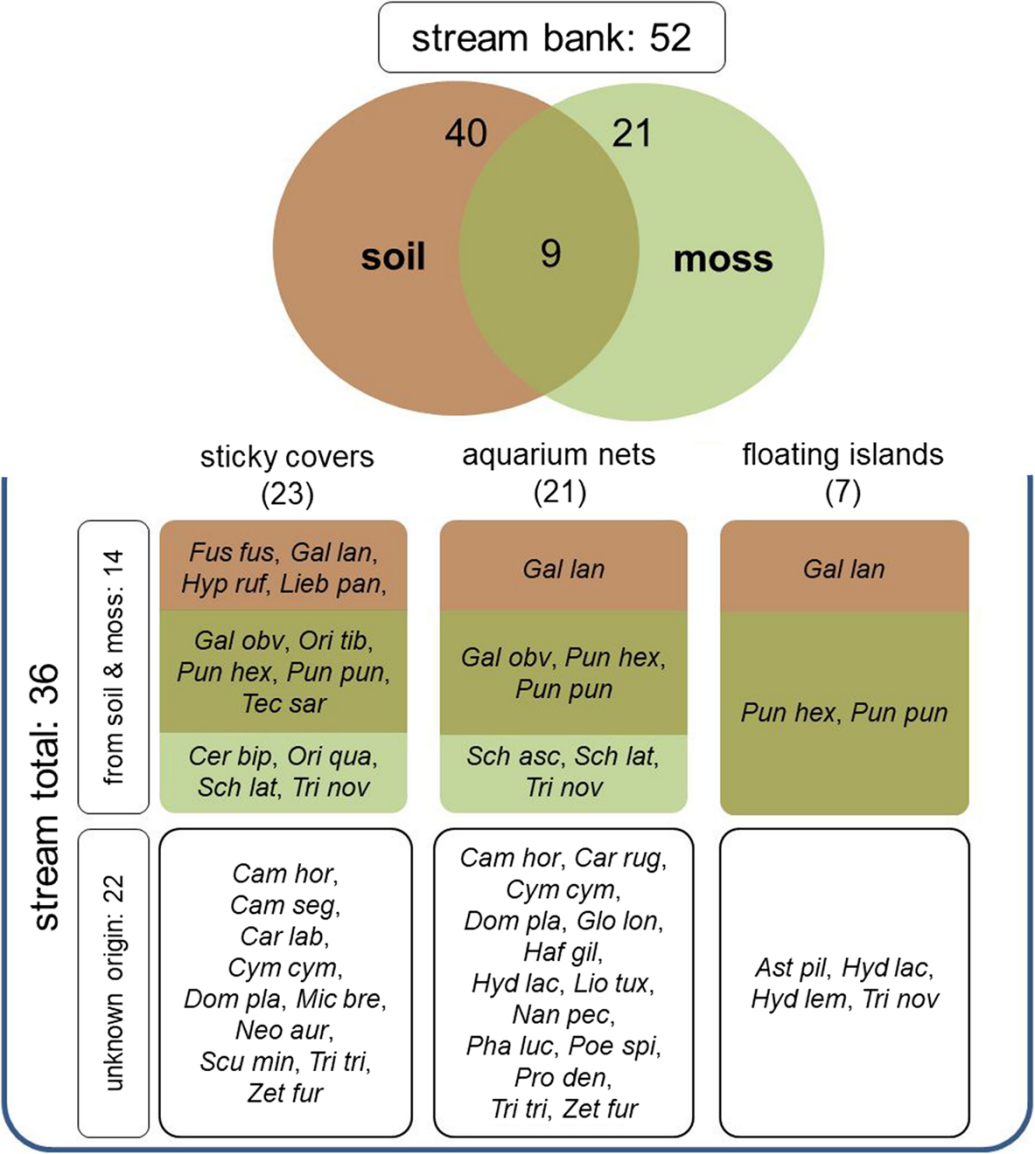
Species numbers recorded along the stream bank and in the stream. Legend: *Ast pil Astegistes pilosus*, *Cam hor Camisia horrida*, *Cam seg Camisia segnis*, *Car lab Carabodes labyrinthicus*, *Car rug Carabodes rugosior*, *Cer bip Ceratoppia bipilis*, *Cym cym Cymbaeremaeus cymba*, *Dom pla Dometorina plantivaga*, *Fus fus Fuscozetes fuscipes*, *Gal lan Galumna lanceata*, *Gal obv Galumna obvia*, *Glo lon Globozetes* cf. *longipilus*, *Haf gil Haffenrefferia gilvipes*, *Hyd lac Hydrozetes lacustris*, *Hyd lem Hydrozetes lemnae*, *Hyp ruf Hypochthonius rufulus*, *Lie pan Liebstadia pannonica*, *Lio tux Liochthonius tuxeni*, *Mic bre Micreremus brevipes*, *Nan pec Nanhermannia pectinate*, *Neo aur Neoribates aurantiacus*, *Ori Qua Oribatella quadricornuta*, *Ori tib Oribatula tibialis*, *Pha luc Phauloppia lucorum*, *Poe spi Poecilochthonius spiciger*, *Pro den Protoribates dentatus*, *Pun hex Punctoribates hexagonus*, *Pun pun Punctoribates punctum*, *Sch asc Scheloribates (Scheloribates) ascendens*, *Sch lat Scheloribates (Scheloribates) latipes*, *Scu min Scutovertex minutus*, *Tec sar Tectocepheus sarekensis*, *Tri nov Trichoribates novus*, *Tri tri Trichoribates trimaculatus*, *Zet fur Zetomimus furcatus*

Altogether, 36 oribatid mite species and 180 individuals were trapped on or in the stream using sticky covers, aquarium nets, and floating islands (Fig. 3). In the soil samples, 5785.2 (± 5292.7) individuals per m^2^ were found, including 40 oribatid mite species (Fig. 3; Additional files 1 and 2). In the moss samples, 21 species of oribatid mites occurred. Altogether, moss and soil samples as a potential source for microarthropods in the stream revealed 52 oribatid mite species occurring, of which only 14 also appeared on or in the stream (Fig. 3; Additional file 1). Twenty-two oribatid mite species that were detected in the stream were not found in the soil or moss samples.

On the sticky covers, 122 individuals and 23 species of Oribatida were found (Fig. 3; Additional file 3), revealing that on average 63.9 (± 54.6) oribatid mite individuals fell on one m^2^ area of water per week. Sticky covers captured only arboricolous and soil-living species (Additional file 1).

In the aquarium nets, 21 oribatid mite species were recorded (Fig. 3; Additional file 4). Only 1.6 (± 4.0) Oribatida individuals per meter cross section of the stream per week were detected, mainly belonging to soil-living and arboricolous species plus two aquatic species. Collembola found on sticky covers could not be identified, but 27 individuals belonging to four terrestrial species were retrieved from the aquarium nets (Additional file 4). Therefore, the mean number of Collembola was 0.9 (± 2.9) individuals per meter cross section of the stream per week.

Floating islands were colonized by five species of terrestrial oribatid mites: *Astegistes pilosus* (C.L. Koch, 1840), *Galumna lanceata* (Oudemans, 1900), *Punctoribates hexagonus* Berlese, 1908, *Punctoribates punctum* (C.L. Koch, 1839) and *Trichoribates* cf. *novus* (Sellnick, 1928) and by four Collembola species: *Desoria trispinata* (MacGillivray, 1896), *Isotomurus plumosus* Bagnall, 1940, *Sminthurides aquaticus* (Bourlet, 1842) and *Sminthurides malmgreni* (Tullberg, 1876) (Additional file 5). Additionally, two aquatic species of Oribatida, *Hydrozetes lacustris* (Michael, 1882) and *Hydrozetes lemnae* (Coggi, 1897), were captured in the islands, whereas arboricolous species were absent. On average, 0.3 (± 0.5) individuals of terrestrial Oribatida and 2.7 (± 5.4) Collembola per island were found (9 individuals of terrestrial Oribatida and 85 Collembola in total). Most abundant was the springtail, *S. aquaticus*, with 69 individuals.

Body size class and sampled habitat were significantly correlated (*p* < 0.01). Species of the first (< 300 μm) and the third size class (> 500 μm) were overrepresented in the stream, while species between 300 and 500 μm were underrepresented here (Table 2). Species larger than 500 μm were underrepresented in the moss samples.

**Table 2:** χ^2^-test (χ^2^ (Pearson) = 153.6 p < 0.01) with individual numbers of oribatids (n) in a certain body size class

		soil	moss	stream	total
< 300 μm	n	3	12	16	31
	expected	6,44	21,48	3,06	
	standardized residuals	–1,35	–2,04 *	7,38 ***	
300-500 μm	n	237	907	67	1211
	expected	251,75	839,43	119,81	
	standardized residuals	–0,92	2.33 *	–4.82 ***	
> 500 μm	n	92	188	75	355
	expected	73,80	246,07	35,12	
	standardized residuals	2,11 *	–3,70 ***	6,72 ***	
	Total	332	1107	158	1597

*significant, **highly significant, ***very highly significant

### Lab studies

**Fig. 4:**
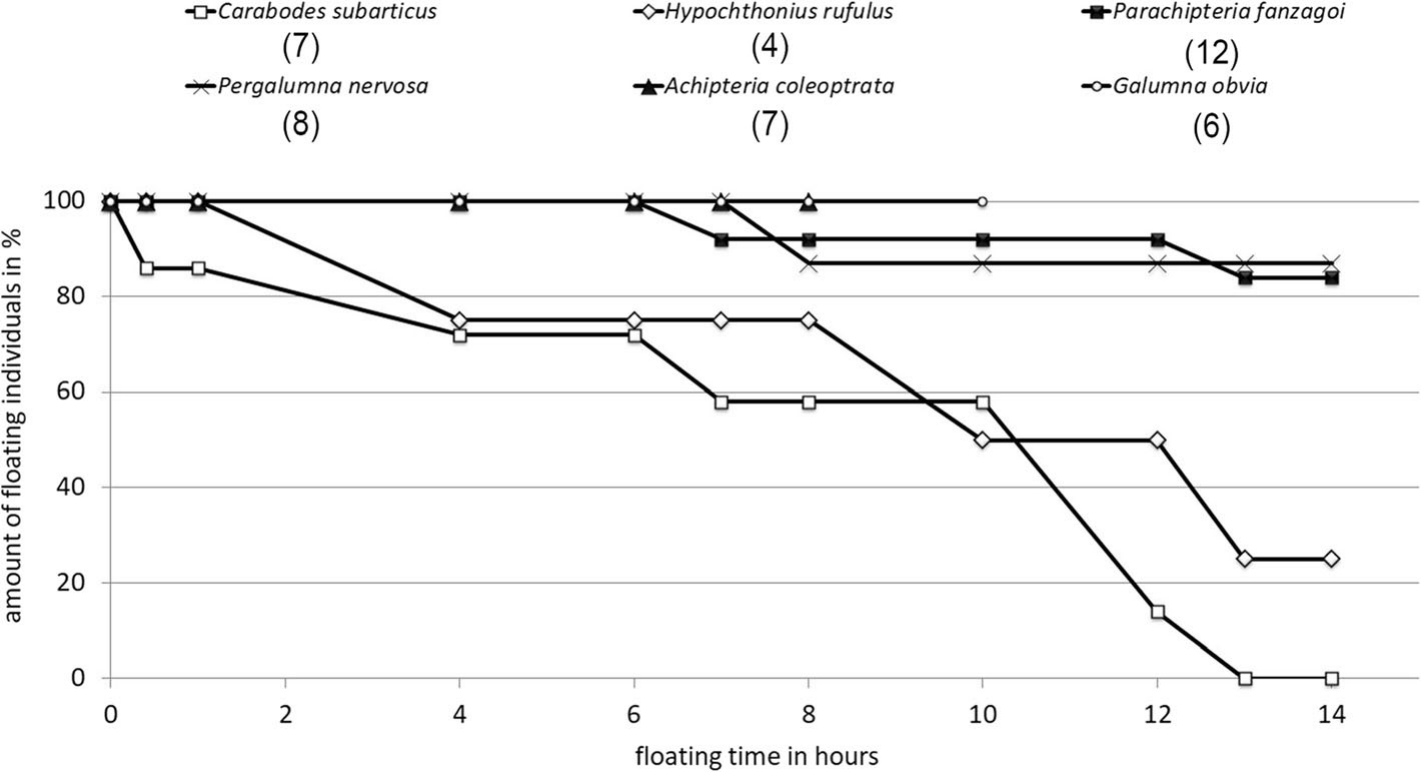
Survival of eleven Oribatida species submerged in fresh water (LT_50_ = Median Lethal Time); numbers behind species names represent numbers of tested individuals

All tested oribatid mite species were able to float on the surface of running water for several hours (Fig. 4). After the 14-h experiment, all individuals of *C. subarcticus* had sunk, whereas some individuals of *P. nervosa*, *P. fanzagoi* and *H. rufulus* were still floating on the water’s surface. These species, therefore, were transported over a distance of approximately 2.5 km when taking into account the velocity of 0.05 m/s in the basin. Floating capabilities were tested for eight hours in *A. coleoptrata*. During that time, all seven individuals remained on the water’s surface and travelled about 1.4 km. Also, in *Galumna obvia* (Berlese, 1914), all six individuals remained on the surface for the ten hours of the experiment (1.8 km). All individuals survived the experiment.

**Fig. 5:**
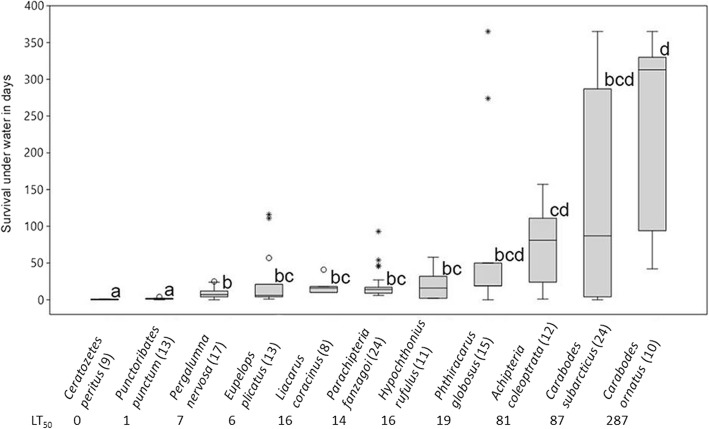
Floating abilities for six studied oribatid mite species; numbers behind species names represent numbers of tested individuals

Survival time under submergence significantly differed between species (H = 79.95, *p* < 0.01). A survival of 365 days was observed in two individuals of *Carabodes subarcticus* Trädgårdh, 1902, two individuals of *Carabodes ornatus* Storkan, 1925, and one individual of *Phthiracarus globosus* (C.L. Koch, 1841) (Fig. 5). Those five individuals were still active when the experiment was stopped after 365 days. Multiplying the maximum survival time and the average velocity of 0.1 m/s of the investigated stream, theoretically they could have been transported approximately 3150 km along a river in 365 days. *Achipteria coleoptrata* (Linné, 1758) also survived tremendous time underwater, with individuals lasting up to 157 days (1356 km). In contrast, *Ceratozetes peritus* Grandjean, 1951, and *P. punctum* survived less than five days, some individuals dying within the first 24 h. Both species survived significantly less time than all other species (*p* < 0.04). *Hypochthonius rufulus* C.L. Koch, 1835, *Pergalumna nervosa* (Berlese, 1914) and *Liacarus coracinus* (C.L. Koch, 1841) survived between one and two months, whereas *Parachipteria fanzagoi* (Jacot, 1929) and *Eupelops plicatus* (C.L. Koch, 1835) survived up to three and four months, respectively (Fig. 5). The LT_50_ value of the investigated species differed between zero and 287 days (Fig. 5). A wide range in survival time was observed between individuals of *A. coleoptrata*, *P. globosus*, *C*. *subarticus*, and *C. ornatus*. Two individuals of *A. coleoptrata* laid eggs after approximately two months of submersion, and the offspring survived for 54 days underwater.

## Discussion

The present study demonstrated for the first time aquatic dispersal of soil microarthropods in running water, including subsequent colonisation of new soil. Based on our results, it is clear that running waters serve as a passive dispersal pathway for terrestrial microarthropods and allow even long-distance dispersal of these otherwise poorly mobile soil organisms. We showed that oribatid mites and Collembola fall into streams, are able to float on the water’s surface, and subsequently colonise new soils. Thereby, successful aquatic dispersal of oribatid mites is facilitated by the ability to float on water for at least several hours and the tremendous submergence tolerance, with survival rates of up to 365 days, a time span that was not published for these organisms before. Even longer submergence survival may be possible because five oribatid individuals from three species were still alive at the end of the experiment.

However, microarthropods are not equal regarding their adaptations to aquatic dispersal. In our floating experiments, 80% of the individuals from four out of six oribatid species floated permanently over the whole time span of the experiment, including all individuals of *A. coleoptrata* and *G. obvia*, but most individuals of *H. rufulus* and all individuals of *C. subarcticus* sank during the 14-h-experiment. Also, the maximum survival times underwater in our study were highly species dependent and ranged from one or two days (*C. peritus*, *P. punctum*) to at least 365 days (*C. subarcticus*, *C. ornatus*, *P. globosus*). High submergence tolerance of oribatid mites has already been proven in previous studies. For example, 76% of all *Camisia anomia* Colloff, 1993 individuals survived 15 days under 6 − 10 °C seawater, a time span which would be sufficient for transport over 700 km with sea currents [74]. Maximum survival rates of intertidal oribatid mite species from the Caribbean Sea, tested at room temperature of 26 ± 1 °C, were approximately 40 days in freshwater and 143 days in saltwater, which would allow transport over 3000 km [53]. In a more recent experiment, the maximum survival time of terrestrial Oribatida submerged in freshwater at 24 °C was 31 days for *G. lanceata* and 165 days for *Platynothrus peltifer* (C.L. Koch, 1839), the 165th day being the end of the experiment [54]. These results were even outperformed by three species in the present experiment. Collembola have also been previously proven to be resistant against submergence, e.g. in *Tetracanthella arctica* Cassagnau, 1959, as 12% of the tested specimens survived a 15-day-experiment at 6-10 °C [52].

Oxygen uptake is indispensable for survival underwater. Intertidal and aquatic oribatid species, like *Hydrozetes*, show plastron capture [75–77], but this is not known for terrestrial oribatid mites [54]. Within the submergence experiment, we could not observe plastron capture either. Hence, respiration at lower O_2_ concentration in water compared to air must take place through the tracheal system, opening into stigmata within the leg region [75, 78–80]. Additionally, lowering the activity reduces the metabolic rate, and therefore the oxygen demand, enhancing the chance of survival underwater [54, 81]. However, the actual effect of submergence tolerance on dispersal success is unknown because it is not clear if a terrestrial microarthropod will be able to get back to land from the bottom of a river and thus be able to colonise a new habitat.

It is possible that the observed egg laying acts as another survival strategy of species under unfavourable conditions, such as during aquatic dispersal [82]. The observation that juveniles of *A. coleoptrata* hatched during submersion is in accordance with findings of successful development of eggs from Acari and Collembola in flooded soil and during submersion experiments [54, 81, 82]. For sexual species like *A. coleoptrata* [83], reproduction would imply fertilization beforehand, while in the many parthenogenetic oribatid species, a new population may originate from just a single individual.

Comparing the observed floating and survival abilities, species could be designated as “swimmers” or “divers”, as suggested by Smrz 1996 [84], who observed immediately sinking species have a much lower mortality rate underwater than “swimmers”. Accordingly, all *C. subarcticus* individuals sank to the bottom during our floating experiment, but survived for 365 days submerged, whereas *P. nervosa* floated quite well for 14 h, but all individuals died within 25 days of submergence. However, most individuals of *A. coleoptrata* and *P. fanzagoi* remained on the water’s surface for 14 h and also tolerated submergence quite well (157 and 93 days maximum, respectively).

From the pool of 52 species extracted from soil and moss samples taken along the stream bank, only 14 species were discovered in the stream. It turned out that the potential to be dispersed by running water obviously depends on body size, with species smaller than 300 μm or larger than 500 μm being overrepresented in the stream. However, it has to be considered that the intermediate size class was dominated by *Zygoribatula exilis* (Nicolet, 1855) with 616 individuals in the moss samples taken from trees. Despite of this high abundance at the stream bank, not a single individual of *Z. exilis* was recorded within the stream. *Zygoribatula exilis* is known to be transported by wind [39] and could also fall into streams, but probably drowns immediately and therefore was not discovered with our sampling method. However, even when the species was excluded from our calculation, species between 300 and 500 μm remained underrepresented in the stream. The influence of body size on aquatic dispersal is exactly the opposite to its influence on wind dispersal [39]: species smaller than 300 μm or larger than 500 μm are rather transported by running water, whereas species of intermediate size are more likely to be transported by wind [39]. Larger and heavier species are probably infrequently transported by wind and rather fall from trees directly onto the surface of the water instead. In soil-living species, the relationship between body size and dispersal may have resulted from their size-dependent horizontal distribution. Species larger than 300 μm are more common on the soil’s surface and in the upper soil layers [85] and therefore more exposed to wind and water than smaller species inhabiting deeper soil layers.

Another reason for the low species congruence between soil and moss samples and the stream may have been that our methods did not detect all species that actually floated on the surface of the stream (see below). Furthermore, specimens may have sunk immediately after falling onto the stream and therefore could not be trapped by the aquarium nets or floating islands. On the other hand, 22 species trapped in the stream were not found in soil and moss samples, most likely because the number of samples was too low to record the entire species pool. On a local scale, soil microarthropod species are usually heterogeneously distributed, restricted by one or more factors, like low movement ability, substrate moisture, resource availability and/or litter diversity [33, 86–89]. For that reason, soil as well as moss samples largely differed in their species composition. Species that were exclusively recorded in the stream, therefore, may have originated from nearby patches that were not sampled or from further upstream, from where they had floated to our sampling sites. Comparing the different methods applied in the stream, species numbers recorded by sticky covers and aquarium nets were much higher than in the floating islands. Hence, many species obviously fell into the stream, but could not colonize new soil after aquatic transport. Particularly for arboricolous species, this is not surprising as floating islands are no suitable habitats for them. However, it is also questionable if all potential colonisers were able to enter the islands or if some of them drifted by.

In addition to body size, further traits like body shape, production of lipid secretions, physiological adaptations, and settled microhabitat conceivably influence water dispersal abilities. However, our preliminary results do not indicate an influence of body shape. Although the body of *H. rufulus* is rather broad and flattened, a shape that could facilitate floating on the water’s surface, three of four individuals had sunk after 13 h of the experiment, and only a single individual was recorded in the stream during the field experiment. In contrast, all individuals of the globular *Galumna obvia* remained on the water’s surface for the entire 10 h of the lab experiment.

*Punctoribates punctum* occurring in soil as well as in moss samples in high numbers, was deposited in the stream from above, floated on the stream’s surface, and colonised one of the floating islands. However, in the submergence experiment, the species did not survive for more than two days. Unfortunately, we had too few individuals to include *P. punctum* in the floating experiment, but at the start of the submergence experiment, it appeared that they could hardly be forced underwater, and their body’s surface seemed very hydrophobic, a characteristic that could well support floating on running water. The anti-wetting effect may have resulted from chemical or physical adaptations [90] because Oribatida and Collembola are known to adapt to varying water contents in soil with the help of a hydrophobic cuticle and cerotegument or lipid secretion [90–93]. The oribatid mite *Liacarus subterraneus* (C.L. Koch, 1844), for instance, is able to produce anti-wetting chemical components mainly composed of carboxyl acids and glycerides after two hours of exposure to water [92]. Also the structure of the outer cement layer and/or the inner cuticle of some oribatid mite species can act as a repellent to water [90]. In Collembola, granules, bristles, and feathered hairs on the body surface act anti-wetting as they allow a plastron creation around the body once the individual is forced to immersion [93]. However, species-specific information about the traits discussed above is very limited, and also the number of species included in our lab experiments was too low to test for the influence of these traits on aquatic dispersal.

We did not observe an influence of the typical habitat, i.e. soil or trees, on aquatic dispersal, because about half of the individuals in the stream were from soil-living species and the other half were arboricolous individuals. Concerning diversity, the number of soil-living species recorded in the stream was higher than the number of arboricolous species; out of 30 species, 21 were represented with a single or a few individuals, usually colonising soil habitats, two species are arboricoles and seven species occur in more than one habitat type [20]. Presumably, arboricolous species are rather transported with branches or leaves drifting on the water’s surface. Furthermore, it was shown that most oribatid mites dispersed by wind are from arboricolous species [39]. In soil-living species, aquatic transport may increase the dispersal success because many soil organisms are sensitive to desiccation and may therefore have better chances to survive aquatic transport compared to wind dispersal.

Nevertheless, several adaptation strategies to aquatic dispersal seem to exist and some species may not be adapted to aquatic dispersal at all. *Hypochthonius rufulus*, for instance, sank relatively quickly and did not survive very long when submerged. Since the species was neither trapped in the aquarium nets nor in the floating islands, it probably uses different dispersal pathways than running waters. Further taxa which were quite abundant in soil and moss samples, like *Minunthozetes semirufus* (C.L. Koch, 1841), *Parachipteria punctata* (Nicolet, 1855), *Protoribates capucinus* Berlese, 1908, and *Z. exilis*, did not appear in the stream at all and, therefore, they might also not be dispersed by water.

Correlating the maximum recorded survival time span of 365 days and the average velocity of 0.1 m/s of the investigated stream, the oribatid mite species *C. subarcticus*, *C. ornatus* and *P. globosus*, may have been theoretically transported around 3150 km along a river. This distance can, however, only be a coarse estimation because seasonality and the related changes in microarthropod density and activity, as well as water temperature, were not considered in the present study. Furthermore, streams and rivers often include barriers and current eddies that prohibit a linear transportation. On the other hand, dispersal distances can be much larger at higher streaming velocities. Nevertheless, for soil organisms with an active dispersal radius of only few centimetres [43, 44], such passive dispersal via running water likely has a significant impact on population dynamics and genetic variation. The high potential of small soil organisms to overcome long distances can explain the wide biogeographical distributions of many species. All terrestrial oribatid mite species found in the floating islands are either known from the whole Palaearctic (*A. pilosus*, *G. lanceata*, *T. novus*) or even the Holarctic region (*Punctoribates hexagonus* Berlese, 1908), or characterized as semi-cosmopolitan (*P. punctum*) [22]. Also, the Collembola found to colonise the floating island are distributed throughout the Palaearctic (*I*. *plumosus*), the Holarctic (*S. malmgreni*), or at least all over Europe (*S. aquaticus*). Especially, *S. aquaticus* was found in high numbers (69 individuals) in the floating islands, suggesting frequent aquatic dispersal.

Running waters could also be a vector for species invasions. The springtail *D*. *trispinata*, in the present study proved to be transported by running water and subsequently colonising new soil, is probably an invader to Europe and was possibly introduced by potting soil [94]. This fast reproducing species can achieve high densities (up to 700,000 ind/m^2^) and was found in water-associated habitats [94]. Hence, aquatic dispersal may have supported its distribution which is almost cosmopolitan today.

Running waters serve as corridors for long-distance dispersal of aquatic species from small streams to the ocean [95, 96]. Our study shows that also terrestrial microarthropods can use these corridors and travel kilometres in a short amount of time. However, the dispersal of aquatic organisms can be inhibited by human regulation of running waters in the form of channelling, hydropower plants, or dam constructions [97–99], and those dispersal barriers most probably also hinder aquatic transport of terrestrial soil invertebrates. All three stages of dispersal, involving departure, transience, and settlement, may be influenced by anthropogenic barriers [100]. Channelling, for instance, may prevent both departure and settlement of a soil microarthropod, as the connection between soil and water is interrupted. Particularly intact floodplains are supposedly important as starting and landing points of soil organisms, but many rivers are highly regulated in their flow regime by straightening and channelling [101–103]. In addition, water-retaining structures may hinder the transition stage of dispersal, but the consequences of barriers to aquatic dispersal on soil microarthropod distribution are unknown so far. However, our results show that tiny terrestrial organisms should not be forgotten when discussing connectivity of systems and long-distance aquatic transport.

The methodological combination of sticky covers, aquarium nets, and floating islands in the field enabled us to distinguish among oribatid mite species that (a) are introduced to a stream by falling from above, (b) actually float on the stream’s surface and (c) can colonise new soil after aquatic dispersal. Although the number of specimens trapped in the floating islands is low, it is sufficient as the first evidence of settlement after aquatic dispersal by soil microarthropods in running water. However, the quantification of transported individuals remains difficult. Specimens on the sticky traps and in the aquarium nets cannot be directly correlated, as the latter method is obviously not appropriate to detect microarthropods on the surface of running waters. Most probably, the low mesh size blocked the passing water, and many individuals just passed by. Moreover, some individuals are slimmer than the available mesh size and might have passed through. Nevertheless, passive dispersal with running water has been proven, but the numbers of floating soil microarthropods may be much higher. Furthermore, our approach only allows an estimation of dispersal distances in the field; efficiency of dispersal and measuring of distance requires additional methods such as population genetics.

## Conclusion

Our study revealed that transport via running waters is obviously a common passive dispersal mechanism of less mobile soil microarthropods. As suggested by the differences in floating abilities and survival of submergence, species may have evolved different adaptations to survive on and underwater. The high floating ability, the enormous submergence survival, and the large number of terrestrial oribatid mites and springtails discovered in the stream point to common long-distance dispersal of these taxa. In addition to the pure transport, we also demonstrated for the first time the potential to colonise new soil after aquatic transport. In future discussions on population dynamics, dispersal strategies, habitat connectivity, and nature conservation, rivers and streams should therefore not only be considered in regard to aquatic organisms, but also to terrestrial soil invertebrates.

## Additional files

Additional file 1: Individual numbers of all oribatid mite species recorded in the field and used in the lab experiments and their main habitat preference after Weigmann et al. 2015. Collembola were only identified from aquarium nets and floating islands; habitat preference are taken from Fjellberg 1998 and 2007. (XLSX 21 kb)

Additional file 2: Species list of oribatid mites found in soil and moss samples and mean numbers of individuals/m^2^. (XLSX 13 kb)

Additional file 3: Species and numbers of Oribatida found on sticky covers. (XLSX 11 kb)

Additional file 4: Species and numbers of Oribatida and Collembola found in the aquarium nets. (XLSX 11 kb)

Additional file 5: Species and numbers of Oribatida and Collembola found in the floating islands. (XLSX 10 kb)

## Data Availability

All data generated or analysed during this study are included in this published article and its supplementary information files.
